# Multiple myeloma burden and risk factors in China vs. high-income Asia Pacific: a macro-micro analysis

**DOI:** 10.3389/fpubh.2026.1784817

**Published:** 2026-03-24

**Authors:** Limin Chai, Yimeng Guo, Linxiu He, Aluo Yang, Liping Su, Jingrong Wang, Jing Yang

**Affiliations:** 1 Department of Pharmacy, Shanxi Province Cancer Hospital/Shanxi Hospital Affiliated to Cancer Hospital, Chinese Academy of Medical Sciences/Cancer Hospital Affiliated to Shanxi Medical University, Taiyuan, China; 2 Department of Hematology, Shanxi Province Cancer Hospital/Shanxi Hospital Affiliated to Cancer Hospital, Chinese Academy of Medical Sciences/Cancer Hospital Affiliated to Shanxi Medical University, Taiyuan, China

**Keywords:** Asia Pacific, China, disease burden, Multiple myeloma, risk factors

## Abstract

**Background:**

Multiple myeloma (MM) presents a growing public health challenge in Asia, yet evidence on its epidemiology and risk factors in transitioning economies like China is limited. This study aimed to compare the disease burden, risk factors, and future trends of multiple myeloma between China and the High-Income Asia Pacific (HIC AP) region.

**Methods:**

We conducted an integrated analysis combining data from the Global Burden of Disease (GBD) study 2023 (1993–2023) with a matched case-control study at a tertiary cancer center in China (2021–2025). The case-control component included 235 newly diagnosed MM cases and 235 healthy controls. Exposures of interest were sex, age, body mass index (BMI), and income level. Main outcomes included age-standardized incidence (ASIR), mortality (ASMR), and disability-adjusted life years (DALYs) rates (ASDR); individual-level risk associations; and Bayesian projections to 2040.

**Results:**

From 1993 to 2023, China's ASIR increased by 175% (from 0.34 to 0.94 per 100 000), while its ASMR plateaued after 2,000. The HIC AP region maintained high but stable ASIRs (2.00 to 2.13 per 100 000) alongside declining ASMRs. In the case-control study, older age (50–69 years: adjusted odds ratio [aOR], 6.69; 95% confidence interval [CI], 4.08–10.99; ≥70 years: aOR, 6.10; 95% CI, 3.30–11.27) and male sex (aOR, 1.59; 95% CI, 1.06–2.37) were significant risk factors. Sex-stratified analysis revealed that higher BMI (24–27.9 kg/m^2^) was associated with increased risk in males (aOR, 2.14; 95% CI, 1.19–3.85) but not in females (aOR, 0.86; 95% CI, 0.45–1.64). Income level showed no significant association. Projections indicate China's ASIR will continue rising to 2.55 per 100 000 by 2,040 with declining ASMR, while the HIC AP burden remains high and stable.

**Conclusion:**

China exhibits transitional MM epidemiology characterized by rapidly rising incidence and plateauing mortality, driven by aging and improved healthcare access. The identified sex-specific association between BMI and myeloma risk highlights population heterogeneity. These findings underscore the need for tailored prevention and control strategies for Asia's evolving disease landscape.

## 1 Introduction

MM is a hematologic malignancy characterized by clonal plasma cell proliferation and monoclonal protein production (1). It represents the second most common hematologic cancer globally, with a well-established geographic disparity: incidence rates are highest in North America and Europe, intermediate in regions like the High-Income Asia Pacific—defined as Japan, Republic of Korea, Singapore, and Brunei Darussalam, and lower but rising rapidly in transitioning economies such as China (2–5). This global pattern underscores the profound influence of socioeconomic development, population aging, and healthcare system maturity on MM epidemiology.

Within Asia, the heterogeneity is particularly instructive. In China, the ASIR of MM increased from 1.47 to 1.74 per 100 000 between 1990 and 2021, reflecting a rapidly growing burden (6). Meanwhile, countries in the HIC AP such as Japan have achieved significant survival improvements, with five–year overall survival rising from 31.2% in 1990–2000 to 50.3% in 2001–2012 (7). Nevertheless, substantial gaps persist between China and HIC AP in early diagnosis, access to innovative therapies, and treatment standardization (8). Notably, a 2025 White Paper revealed high misdiagnosis rates (47.3%) for myeloma bone disease in China, underscoring systemic challenges in primary care and imaging utilization (9).

The contrasting yet adjacent epidemiological settings of China and HIC AP thus create a powerful, naturally occurring comparative framework. Both regions face accelerating population aging, yet they reside at distinct points along the spectrums of socioeconomic development and health system maturation (10). This study leverages these systematic developmental disparities to establish an analytical framework to explore the divergent evolution of multiple myeloma burden between the two settings. Through this framework, we aim to identify modifiable drivers and actionable intervention points, thereby generating empirical evidence to inform the design of prevention and control strategies in health systems undergoing similar transitions.

However, existing studies remain largely descriptive or confined to single countries, lacking integrative analyses that bridge macro-level burden trends with micro-level risk factors validated in real-world populations. To address this gap, we established a “macro-micro” bidirectional validation framework. Using GBD2023 data, we analyzed MM incidence, mortality, and DALYs trends in China and HIC AP from 1993 to 2023. We then examined the individual-level associations of four macro-identified factors-age, sex, BMI, and income-with MM risk through a case-control study (235 matched pairs) in China. This integrated approach enhances causal inference and provides a robust epidemiological basis for predictive modeling and targeted prevention strategies.

## 2 Materials and methods

### 2.1 Data sources

Data on MM incidence, mortality, DALYs, and ASIR, ASMR, ASDR for China and HIC AP from 1993 to 2023 were obtained from the GBD 2023 study via the Global Health Data Exchange (GHDx).

### 2.2 Study design and participants

This study employed an exploratory hospital-based case-control design to bridge macro-level trends with micro-level evidence. The study was conducted at the Shanxi Province Cancer Hospital, with a participant enrollment period from January 2021 to December 2025. Case Group: we consecutively enrolled all newly diagnosed, pathology- and immunofixation-confirmed symptomatic MM patients (age ≥18 years) within the study period. Patients with all common immunochemical subtypes (e.g., IgG, IgA, IgM, light chain, and others) were included. Exclusion criteria included monoclonal gammopathy of undetermined significance, smoldering multiple myeloma, secondary plasma cell dyscrasias, and individuals with missing key clinical data. A total of 235 patients were included. Control Group: controls were selected from individuals undergoing routine health examinations at the same institution during the same period. To ensure representativeness of the source population and minimize selection bias, simple random sampling was employed: a list of all eligible examinees (with no history of malignancy, age ≥18 years) was obtained, and individuals were selected using computer-generated random numbers until the sample size matched the case group (*n* = 235).

### 2.3 Risk factors and analysis

This study employed a “macro-guided, micro-analyzed” strategy for risk factor selection. The macro-level analysis of GBD 2023 data revealed three key patterns: ➀ distinct age and sex distribution patterns in MM burden; ➁ high BMI as the only behavioral/metabolic risk factor formally quantified and attributable to MM mortality in the GBD framework, with divergent evolution of its attributable burden between the two regions; and ➂ region (reflecting socioeconomic and environmental disparities) as a critical stratification variable for burden differences. Based on this macro-level evidence, we selected four factors for individual—level analysis in a case—control study: sex; age (categorized as < 50, 50–69, or ≥70 years); BMI (categorized as < 24.0, 24.0–27.9, or ≥28.0 kg/m^2^ according to Chinese criteria) (11); and income level (dichotomized at the national median disposable income of CNY 27,149) (12). All data were extracted from the hospital's electronic medical records system and verified independently by two researchers to ensure accuracy.

### 2.4 Sample size consideration

The sample size was determined through a dual approach combining prospective estimation and sensitivity power analysis. During the design phase, a formal sample size calculation was conducted to establish a methodological benchmark. Given that age represents an overwhelmingly strong risk factor for MM (typically OR >5), using it as the basis for estimation would have yielded an inappropriately small sample size, insufficient for evaluating other clinically relevant factors with more moderate effect sizes, such as sex and BMI. Therefore, sex (male vs. female) was selected as the calculation benchmark due to its well-documented, stable association with MM risk globally. With a target odds ratio of 1.6, a control group male proportion of 0.5, and standard statistical parameters (α = 0.05, two-tailed; power = 80%), the prospective calculation indicated a theoretical requirement of approximately 287 pairs (574 subjects). In practice, the final sample size was determined by the prespecified 60-month (5-year) enrollment period, guided by the principle of securing a complete and high-quality case series through systematic ascertainment. Through consecutive enrollment of all eligible incident MM cases and simple random sampling of matched healthy controls over this period, the study achieved a final analytic sample of 235 pairs (470 subjects). This represents the largest systematically ascertained, high-quality case series for MM from this center within a defined multi-year framework. To objectively evaluate the effect size detectability given the achieved sample, a sensitivity power analysis was performed using G^*^Power (version 3.1). With a total sample of 470 subjects, α = 0.05 (two-tailed), and 80% power, the minimum detectable odds ratio was 1.65.

### 2.5 Statistical analysis

All statistical analyses were performed using SPSS (version 27.0) and R software (version 4.5.2). Temporal trends in age-standardized disease burden metrics from 1993 to 2023 were analyzed using Join point regression to estimate the average annual percentage change. Associations between individual-level risk factors and MM were assessed via multivariable binary logistic regression; all variables (sex, age, BMI, income) were included simultaneously to control for confounding, with results reported as adjusted odds ratios (aORs) and 95% confidence intervals (CIs). To examine potential effect modification, a “sex × BMI” interaction term was tested within the model, and stratified analyses were conducted accordingly. Future age-standardized incidence and mortality rates (2024–2040) were projected using the Bayesian Age-Period-Cohort model. It is noted that results originating from the GBD study are presented with 95% uncertainty intervals, while those from the present case-control study are reported with 95% CIs.

### 2.6 Ethics statement

The study protocol was reviewed and approved by the Ethics Committee of Shanxi Province Cancer Hospital (Approval No.: KY2026001).

## 3 Results

### 3.1 Overall trends in incidence, mortality, and DALYs

**Figure 1:**
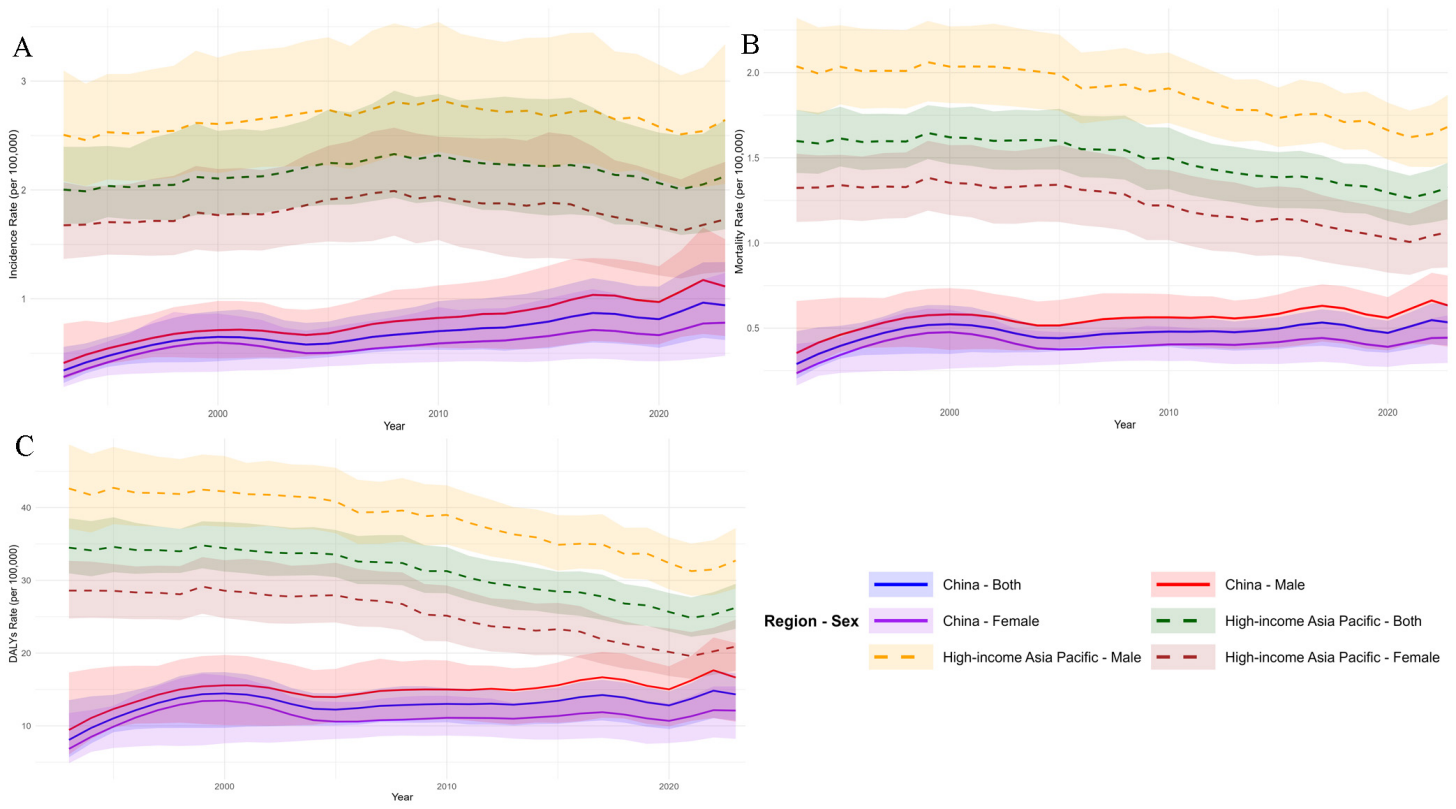
Sex-specific and overall trends in age-standardized rates for multiple myeloma in China and high-income Asia Pacific, 1993–2023. **(A)** Incidence, **(B)** Mortality, and **(C)** DALY rates for China (solid lines) and HIC AP (dashed lines). Legend: China-overall **(blue)**, Male **(red)**, Female **(purple)**; HIC AP-Overall (green), Male **(yellow)**, Female **(burgundy)**. The *x*-axis indicates year (1993–2023); the *y*-axis indicates rate per 100 000 population (age-standardized). Shaded areas: 95% uncertainty intervals. Data are from the Global Burden of Disease Study 2023.

Between 1993 and 2023, MM burden diverged markedly between China and HIC AP. China's ASIR increased by 175% (0.341 to 0.940 per 100 000), while HIC AP's ASIR remained high but stable (2.003 to 2.125). Mortality and DALY trends also differed: China's rates plateaued after 2,000, whereas HIC AP's declined steadily (Table 1, Figure 1).

**Table 1:** All age cases and age standardized incidence, mortality, and DALY rates with corresponding AAPC for multiple myeloma in China and high income Asia Pacific, 1993 and 2023.

**Location**	**Measure**	**Year**	**All-age cases (95% UI)**	**Age-standardized rate per 100 000 (95% UI)**	**AAPC, 1993–2023 (95% CI)**
China	Deaths	1993	2,756.37 (1,941.19 to 4,598.83)	0.29 (0.20 to 0.48)	2.027 (0.959 to 3.106)
		2023	12,203.70 (9,094.52 to 14,783.24)	0.53 (0.40 to 0.64)	
	Incidence	1993	3,363.40 (2,218.60 to 5,479.97)	0.34 (0.23 to 0.56)	3.441 (2.447 to 4.414)
		2023	21,574.43 (14,296.28 to 30,466.87)	0.94 (0.62 to 1.34)	
	DALYs	1993	85,063.71 (59,315.67 to 142,236.53)	8.07 (5.67 to 13.53)	1.912 (0.954 to 2.878)
		2023	320,550.16 (237,321.38 to 384,497.40)	14.32 (10.70 to 17.22)	
HIC AP	Deaths	1993	3,473.47 (3,080.80 to 3,870.80)	1.60 (1.41 to 1.78)	−0.583 (−0.920 to −0.244)
		2023	7,707.72 (6,394.20 to 8,651.75)	1.33 (1.15 to 1.49)	
	Incidence	1993	4,393.98 (3,711.65 to 5,252.61)	2.00 (1.69 to 2.40)	0.208 (−0.191 to 0.609)
		2023	11,277.17 (8,664.66 to 13,736.23)	2.13 (1.64 to 2.64)	
	DALYs	1993	76,840.89 (69,183.56 to 85,862.67)	34.49 (30.97 to 38.52)	−0.904 (−1.264 to −0.543)
		2023	127,411.16 (109,915.88 to 143,071.59)	26.23 (23.34 to 29.51)	

AAPC, average annual percentage change; CI, confidence interval; DALY, disability-adjusted life year; HIC AP, High-Income Asia Pacific; UI, uncertainty interval.

### 3.2 Age-stratified differences

Age-specific analysis revealed that the rising incidence of MM in China was primarily driven by the middle-aged and elderly populations. The most pronounced absolute increase occurred in the ≥70 years age group, where the incidence rate surged from 2.12 to 5.71 per 100 000. The 50–69 years age group also showed a marked rise from 1.07 to 2.92 per 100 000. Consistent with this trend, age-specific mortality rates (ASpMR) and age-specific DALY rates (ASpDR) increased significantly across all age groups in China, with the ≥70 years group seeing mortality rise from 2.05 to 3.96 per 100 000.

**Figure 2:**
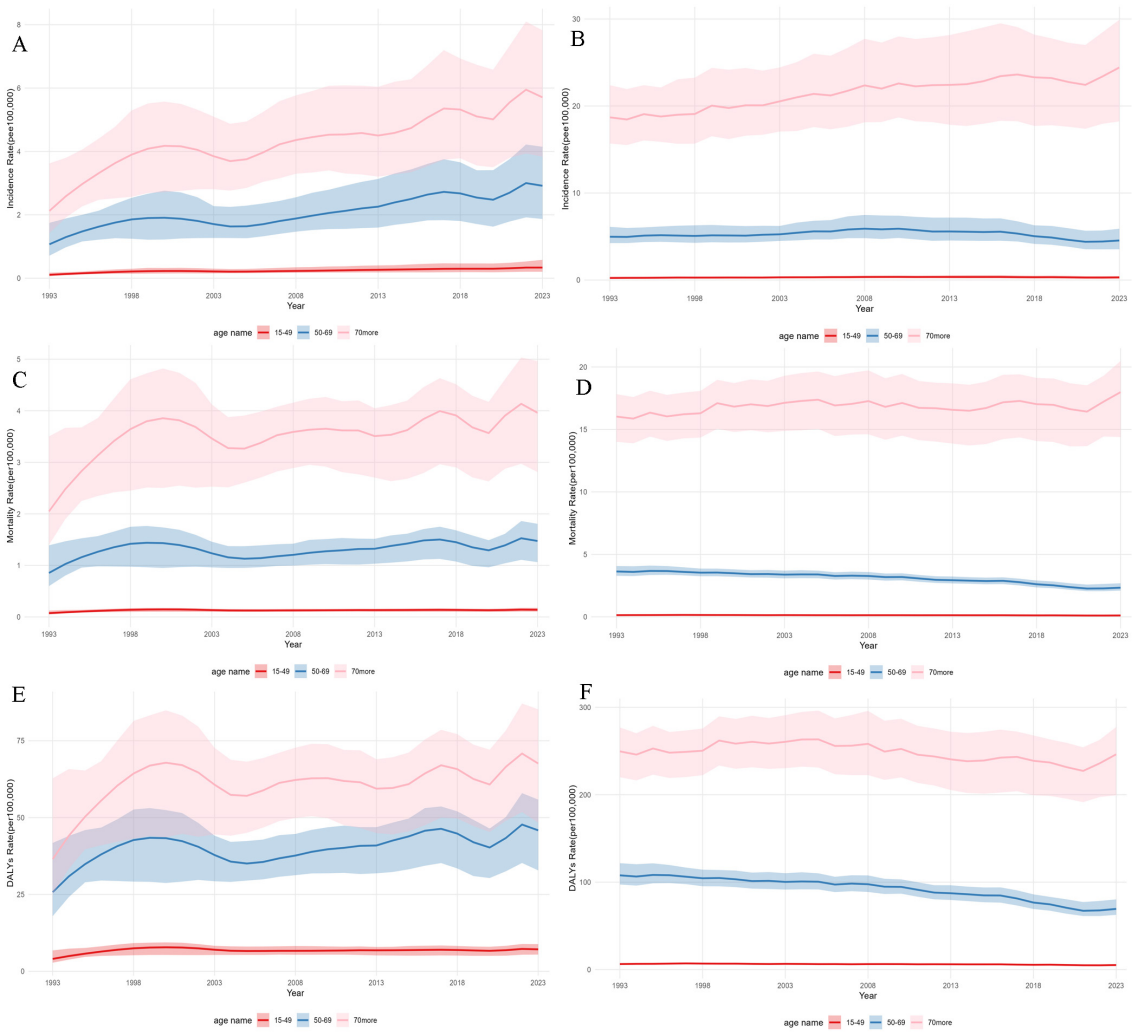
Age-specific incidence, mortality, and disability adjusted life years rates of multiple myeloma in China and high-income Asia Pacific, 1993–2023. China **(A, C, E)** and HIC AP **(B, D, F). (A, B)** Incidence; **(C, D)** Mortality; **(E, F)** DALY rates. Lines: 15–49 years **(red)**, 50–69 years **(blue)**, ≥70 years **(pink)**. Axes: *x*-axis indicates year (1993–2023); *y*-axis indicates rate per 100 000 population (agespecific). Shaded areas: 95% uncertainty intervals. Data are from the Global Burden of Disease Study 2023.

In contrast, in the HIC AP, the incidence in the 50–69 years group experienced a gradual decline from 4.97 to 4.54 per 100 000. Moreover, both mortality and DALYs across all age groups in this region exhibited significant declines or remained stable. Notably, the incidence in the HIC AP ≥70 years group remained exceptionally high, increasing from 18.70 to 24.43 per 100 000–approximately 4.3 times higher than in China's comparable age group by 2023. However, the mortality rate in this same HIC AP age group decreased from 16.03 to 17.99 per 100 000, having reached even lower levels during the interim period (Figure 2). In both regions, the population aged ≥70 years constituted the absolute majority of the disease burden.

### 3.3 Sex differences

A consistent male predominance in disease burden was identified in both regions. In China for the year 2023, the incidence and mortality rates among males (1.114 and 0.624 per 100 000, respectively) were approximately 1.4 times those of females (0.781 and 0.445 per 100 000). A similar significant sex disparity was maintained in the HIC AP throughout the study period.

In summary, the epidemiological profiles of the two regions are distinct: China is in a transitional phase, characterized by rapidly rising incidence across all adult age groups, while mortality and overall burden have entered a plateau phase. The HIC AP exhibits a mature control profile, with a high but stable overall incidence, coupled with effectively declining mortality and disease burden.

### 3.4 Trends in mortality burden attributable to high BMI

China and HIC AP exhibited significant differences in the evolutionary pathways and driving factors of mortality burden related to high BMI. The ASMR for related diseases in China has entered a high plateau phase, yet the proportion of mortality burden attributable to high BMI continues to grow strongly. In contrast, HIC AP countries, despite having a higher baseline mortality rate, have achieved a sustained and gradual decline in overall mortality. The proportion of high BMI-attributable burden in these regions shows only a modest increase and remains stable.

**Figure 3:**
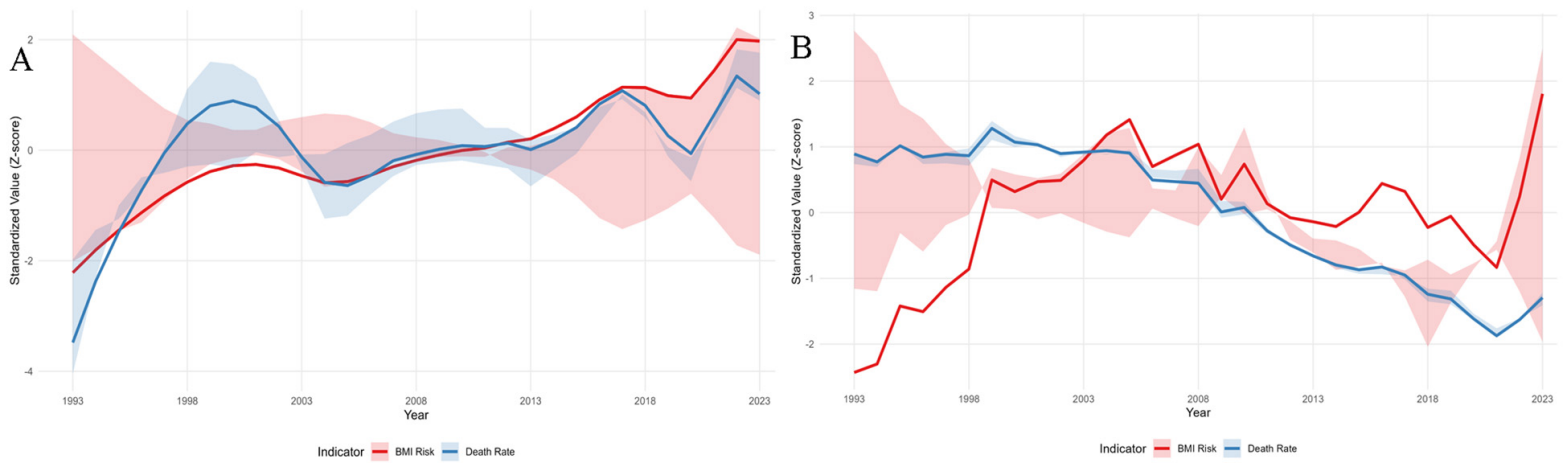
Standardized trends of high BMI-attributable and overall mortality from multiple myeloma in China and high-income Asia Pacific, 1993–2023. **(A)** China. **(B)** High income Asia Pacific (HIC AP). **Red** lines indicate mortality attributable to high BMI; **blue** lines indicate overall age standardized mortality rate. The *y*-axis indicates standardized value (Z score), where 0 represents the mean across the study period. The *x*-axis indicates year (1993–2023). Shaded areas represent 95% uncertainty intervals. Data are from the Global Burden of Disease Study 2023.

In summary, the epidemiological characteristics of the two regions are distinct: China is in a transitional phase, characterized by rapidly rising incidence rates across all adult age groups, while mortality rates and overall burden have entered a plateau. The HIC AP exhibits a mature control profile, with a high but stable overall incidence, coupled with effectively declining mortality and disease burden (Figure 3).

### 3.5 Risk factors analysis

To examine whether the association between BMI and MM risk was modified by sex, we included a “sex × BMI” interaction term in the multivariable logistic regression model adjusted for age and income. The global test for interaction was statistically significant (Wald χ^2^ = 9.305, df = 2, *p* = 0.010) (Table 2). Specifically, the interaction term for male × overweight (BMI 24–27.9 kg/m^2^) yielded an OR of 1.99 (95% CI: 1.27–3.11, *p* = 0.003), indicating that the effect of overweight on MM risk was significantly stronger in males than in females. No significant interaction was observed for male × obesity (BMI ≥28 kg/m^2^) (OR = 1.39, 95% CI: 0.62–3.08, *p* = 0.424). Based on these findings, we performed sex-stratified analyses to further elucidate the risk factor profiles in males and females separately (Table 3).

**Table 2:** Multivariable binary logistic regression analysis of MM.

**Variable**	**Category (reference)**	**β coefficient**	**SE**	**Wald χ^2^**	***P* value**	**aOR (95% CI)**
Sex				5.128	0.024	
	Male (vs. Female)	0.463	0.205			1.589 (1.064–2.373)
Age (years)				59.312	< 0.001	
	50–69 (vs. < 50)	1.901	0.253	56.503	< 0.001	6.693 (4.077–10.988)
	≥70 (vs. < 50)	1.808	0.313	33.336	< 0.001	6.100 (3.302–11.270)
BMI (kg/m^2^)				2.914	0.233	
	24–27.9 (vs. < 24)	0.337	0.216	2.423	0.120	1.400 (0.916–2.139)
	≥28 (vs. < 24)	0.373	0.321	1.354	0.245	1.452 (0.775–2.721)
Income level				1.223	0.269	
	High (vs. Low)	0.246	0.222			1.279 (0.827–1.978)
**Interaction effects**						
Sex × BMI				9.305	0.010	
	Male × BMI 24–27.9 (vs. Female × BMI < 24)	0.688	0.228	9.132	0.003	1.990(1.272–3.114)
	Male × BMI ≥28 (vs. Female × BMI < 24)	0.326	0.408	0.640	0.424	1.386 (0.622–3.084)
Constant		−1.895	0.289	43.121	< 0.001	0.150

Model statistics: omnibus test χ^2^ = 78.856, *p* < 0.001; Hosmer—Lemeshow test χ^2^ = 2.334, *p* = 0.969; Nagelkerke *R*^2^ = 0.206; Overall correct classification = 66.4%. aOR, adjusted odds ratio; BMI, body mass index; CI, confidence interval; SE, standard error.

**Table 3:** Stratified multivariable logistic regression analysis by sex.

**Variable**	**Subgroup**	***P* value**	**aOR (95% CI)**
Age (vs. < 50)	Male (50–69)	< 0.001	7.029 (3.624, 13.633)
	Female (50–69)	< 0.001	6.461 (3.021, 13.817)
	Male (≥70)	< 0.001	11.136 (4.618, 26.856)
	Female (≥70)	0.010	3.387 (1.332, 8.612)
BMI (vs. < 24)	Male (24–27.9)	0.012	2.135 (1.185–3.849)
	Female (24–27.9)	0.640	0.857 (0.449–1.636)
	Male (≥28)	0.211	1.843 (0.707–4.802)
	Female (≥28)	0.927	1.041 (0.440–2.468)
Income level (vs. < low)	Male (24–27.9)	0.147	1.546 (0.858–2.785)
	Female (24–27.9)	0.615	0.839 (0.423–1.664)

Effect size interpretation based on sensitivity power analysis: based on the sensitivity power analysis, the minimum detectable OR for this study at 80% power was 1.65 (see Methods 2.4). The effect sizes for age (OR = 6.69 and 6.10) and the male-specific overweight effect (OR = 2.14) substantially exceeded this threshold, indicating excellent statistical power and high robustness. The male sex effect (OR = 1.59) fell slightly below the threshold (corresponding power ≈78%), yet remained statistically significant (*p* = 0.024) with a 95% CI lower bound (1.06) above the null value, suggesting a genuine but modest association.

The multivariable logistic regression was used to assess the associations between sex, age, BMI, and income level with the risk of MM. The results indicate that sex and age are independent influencing factors for MM, with BMI also showing a significant association specifically among males.

In the overall sample (*n* = 470), age emerged as the strongest predictor of MM. Compared to age < 50 group (reference), the risk was significantly higher in both 50–69 age group [adjusted odds ratio (aOR) = 6.69, 95% CI: 4.08–10.99] and ≥70 age group (aOR = 6.10, 95% CI: 3.30–11.27) (both *p* < 0.001). Males exhibited a significantly higher risk than females (aOR = 1.59, 95% CI: 1.06–2.37, *p* = 0.024). No significant associations were observed for BMI or income level in the overall sample (Table 2). Subgroup analysis showed that higher BMI (24–27.9 vs. < 24) was significantly associated with an increased risk of MM in males (*n* = 260; aOR = 2.14, 95% CI: 1.19–3.85, *p* = 0.012), whereas no significant association was observed in females (*n* = 210) (Table 3). In summary, our results indicate that age is the most consistent risk factor for MM, with males having a significantly higher risk. BMI was independently associated with MM risk, but only in the male population.

### 3.6 Future burden of MM projections in China and HIC AP over the next 15 years

**Figure 4:**
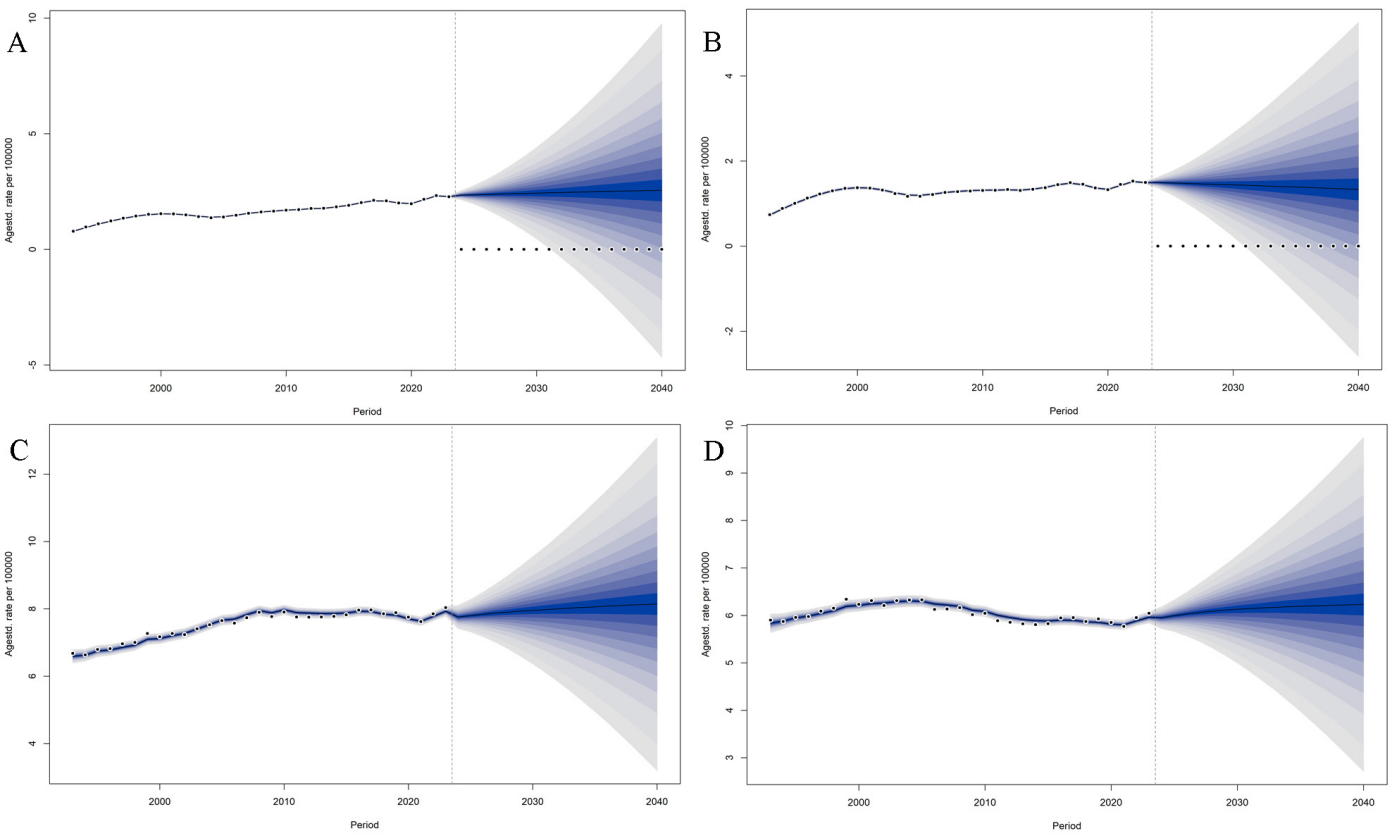
Observed and projected age-standardized incidence and mortality rates of multiple myeloma in China and high-income Asia Pacific, 1993–2040. **(A)** Incidence in China. **(B)** Incidence in high income Asia Pacific (HIC AP). **(C)** Mortality in China. **(D)** Mortality in HIC AP. Solid lines represent observed data (1993–2023); dashed lines represent projected data (2024–2040). The vertical line at 2023 indicates the transition point. Rates are per 100 000 population (age standardized). Source: Observed data from GBD 2023; Projections based on Bayesian age-period-cohort models.

The forecast results for 2024–2040 indicate that China's ASIR is projected to increase gradually from 2.35 to 2.55 per 100 000 people, with an average annual increase of approximately 0.006. In the same period, the ASMR is expected to decline steadily from 1.50 to 1.33 per 100 000 people. In contrast, the HIC AP shows ASIR rising from 7.76 to 8.14 per 100 000 people, with an average annual increase of about 0.022, and ASMR gradually increasing from 5.95 to 6.23 per 100 000 people. By 2040, the ASIR in the HIC AP remains 3.2 times that of China, while ASMR is approximately 4.7 times higher. A comparison between the two regions reveals a pattern of “slowly rising incidence and slowly declining mortality” in China, whereas the HIC AP exhibits a steady and gradual increase in both indicators. Throughout the forecast period, the absolute gap in disease burden between China and the HIC AP remains significant (Figure 4).

## 4 Discussion

This study integrates macro-level disease burden data with micro-level clinical evidence to provide the first systematic analysis of the differences in the disease burden evolution and risk factors of MM between China and HIC AP over the past three decades, along with predictions of future trends.

### 4.1 Integrated analysis of disease burden trends and future projections

Our integrated analysis reveals a distinct “catch-up and decoupling” trajectory in China's MM epidemiology over the past three decades. The 175% rise in ASIR reflects synergistic drivers: systematic improvements in diagnostic infrastructure (e.g., specialized hematology networks and serum protein electrophoresis) (13–15), rapid population aging expanding the high-risk pool, and enhanced treatment accessibility. This is reflected in: (i) sequential inclusion of bortezomib, lenalidomide, and daratumumab in the National Reimbursement Drug List, substantially reducing out-of-pocket costs; (ii) continuous updates of the Chinese Society of Hematology MM guidelines with nationwide dissemination through academic programs and standardized care demonstration centers; and (iii) a 15-year single-center study of 1,256 MM patients in China demonstrating progressive improvements in response rates, PFS, and OS, driven by wider adoption of novel agents and advanced diagnostic techniques (15). This therapeutic progress underlies the observed plateau and gradual decline in ASMR since 2000, signaling a positive shift toward chronic disease management. However, compared to the consistent mortality decline in HIC AP, China's stabilization suggests that diffusion of innovative therapies and standardization of care remain uneven. Notably, the growing proportion of MM mortality attributable to high BMI in China underscores obesity as an emerging obstacle to further mortality reduction-a challenge that HIC AP has mitigated through more efficient medical interventions and comprehensive risk management.

Looking forward, our projections indicate that China's ASIR will continue to rise gradually (reaching 2.55 per 100 000 by 2040), while ASMR declines, further widening the “incidence-mortality gap.” This trend confirms the positive impact of advancing diagnostics and treatment, but also warns of sustained pressure on healthcare systems, especially in geriatric and hematology services. In contrast, HIC AP exhibits a pattern of high-level stabilization, with both incidence and mortality projected to remain elevated but stable. The narrowing burden gap between the two regions reflects China's progress while signaling its transition toward a developed-region disease profile. This underscores the urgent need for China to adopt forward-looking, dual-focused strategies: continuing to optimize standardized diagnosis and treatment to consolidate mortality gains, while elevating primary prevention to a strategic level-particularly through population-health initiatives targeting modifiable risks such as obesity, especially among overweight males.

### 4.2 Analysis of risk factors

Through real-world case-control analysis, this study confirmed advanced age as the most powerful risk factor for MM (50–69 years: aOR = 6.69; ≥70 years: aOR = 6.10), consistent with its well-established nature as an age-related malignancy (3, 16, 17). The effect sizes far exceeded the minimum detectable OR of 1.65 at 80% power (see Methods 2.4), demonstrating excellent statistical robustness despite the modest sample size. Male sex was also an independent risk factor (aOR = 1.59, 95% CI: 1.06–2.37), corroborating global epidemiologic data showing a male-to-female incidence ratio of approximately 1.5:1 (18, 19). This effect size fell slightly below the detectable threshold (corresponding power ≈78%), yet it remained statistically significant with a 95% CI lower bound above unity, supporting a genuine but modest association. The male predominance in MM risk may reflect sex-chromosome-related genetic predisposition, hormone-mediated differences in the immune microenvironment, or distinct behavioral patterns (20–22).

This study specifically investigated the relationship between BMI and the risk of developing MM. Results showed no significant association between BMI and MM risk in the overall sample. However, sex-stratified analysis revealed that higher BMI levels were significantly associated with an increased risk of MM in males, while no similar association was observed in females. This finding differs from conclusions drawn in Western studies and by the International Agency for Research on Cancer (IARC) (23–25). IARC has classified high BMI as a confirmed risk factor for MM, and mechanisms, through which it promotes MM, such as chronic inflammation and metabolic dysregulation, have been elucidated (26). Concurrently, this sex-specific pattern shows inconsistencies within similar Asian studies. For instance, a prospective study involving 780,000 Korean males and the Japan Public Health Center-based Prospective Study (JPHC) reported no significant association between BMI and incidence (27, 28). The association between BMI and MM risk exhibits evident population heterogeneity among Asian populations, particularly showing a sex-dimension pattern different from Western studies. This divergence may reflect biological differences between Eastern and Western populations in body composition, chronic inflammatory pathways, and genetic susceptibility (29, 30). Therefore, when formulating MM prevention strategies for Asian populations, it is crucial to fully consider the population-specific and sex-specific effects of risk factors and to promote subsequent research to elucidate their biological foundations from genetic, metabolic, and molecular perspectives.

### 4.3 Socioeconomic factors

In the individual-level analysis of this study, no significant association was found between income level and MM risk, suggesting that economic factors *per se* may not be key risk factors for the disease within the specific region studied. This finding can be attributed to two main reasons: first, under conditions of regionally strong environmental exposure (such as in the industrial province included in this study), widespread environmental risk factors may override individual socioeconomic differences and become the dominant source of disease causation. This hypothesis is directly supported by recent epidemiological evidence. A 2025 multicenter case-control study from Palestine (227 MM cases, 176 matched controls) reported that exposure to cosmetics-related chemicals was associated with a nearly three-fold increased risk of MM (OR = 2.85; 95% CI: 1.56–5.21); pesticide exposure also showed a positive association (31). Furthermore, a pooled analysis by the International Multiple Myeloma Consortium comprising five international case-control studies (1,959 cases, 6,192 controls) found a 50% elevated risk among gardeners and nursery workers (likely exposed to pesticides) (OR = 1.50; 95% CI: 0.9–2.3), and moderately increased risks for metal processors, female cleaners, and high-level organic solvent exposure (32). These findings, consistent with our observations, collectively underscore that occupational pesticide, organic solvent, and specific chemical exposures are modifiable risk factors for MM that cannot be neglected. Second, China's medical security system, particularly the universal health coverage and primary healthcare service system, plays a significant role in mitigating and buffering health inequalities stemming from differences in individual payment capacity. This indicates that the influence of income on disease risk is substantially moderated by regional environmental characteristics and health policy systems, highlighting the complexity and context-dependency of social determinants of health. Further studies conducted under different institutional and environmental contexts are warranted to clarify the specific pathways involved.

## 5 Limitations

The primary strength of this study lies in its adoption of an integrated “macro-micro” validation framework, which combines long-term trend data, predictive modeling, and regional real-world clinical evidence, thereby enhancing the robustness of its conclusions. The limitations of the study include the following: reliance on GBD model-estimated data, whose quality is contingent upon the underlying source data; the use of a clinical sample from a single center, which may limit the generalizability of the findings; the absence of key variables such as genetic factors and specific environmental exposures, such as occupational exposures (e.g., to agricultural chemicals, benzene), air pollution, or dietary patterns; a study population primarily comprising newly diagnosed surviving cases, resulting in lack of the analysis of mortality-related risk factors and necessitates subsequent follow-up studies for completion; and the constraints of the predictive model, which is based on historical data and thus has limited ability to prospectively account for potential breakthroughs in treatment or major public health interventions.

Additionally, the achieved sample size (235 pairs) fell short of the prospectively estimated target (287 pairs). Consequently, the statistical power for detecting a moderate effect size—such as the male sex effect (OR = 1.59)—was slightly below the conventional 80% threshold (actual power ≈78%). Nevertheless, this association was still statistically significant, and its 95% CI lower bound exceeded 1.0, supporting its validity. For effect sizes of larger magnitude (age, male-specific BMI effect), the study was adequately powered and the conclusions are robust. Future multi-center studies with larger samples are warranted to validate these findings and to explore the underlying biological mechanisms.

## 6 Conclusion

This study systematically reveals the “catch-up and decoupling” characteristic of multiple myeloma in China, characterized by a rapid rise in incidence alongside a plateauing mortality trend. It analyzes the population-specific nature of risk factors and projects a continued increase in future disease burden. The findings underscore the need to establish a comprehensive prevention and control system that integrates treatment with prevention and shifts the focus toward early intervention. Addressing this public health challenge requires both optimizing clinical management and strengthening primary prevention strategies.

## Data Availability

The data analyzed in this study is subject to the following licenses/restrictions: Access to the clinical data from Shanxi Provincial Cancer Hospital is restricted to protect patient privacy. Sharing is prohibited by the hospital's Ethics Review Board approval. De-identified data may be available to researchers upon reasonable request to the corresponding authors (Jing Yang or Jingrong Wang), subject to approval by the Ethics Board and a data use agreement. Requests to access these datasets should be directed to isyangjing@sxmu.edu.cn
